# Understanding Device Integrations Within Diabetes Apps: Mixed Methods Analysis of App Features and User Reviews

**DOI:** 10.2196/62926

**Published:** 2025-05-27

**Authors:** Jessica Turner, Katarzyna Stawarz

**Affiliations:** 1 University of Waikato, 101 Durham Street, Tauranga, 3110, New Zealand, 64 7 262 0585; 2 Cardiff University, Cardiff, United Kingdom

**Keywords:** diabetes mellitus, health apps, mHealth, mobile health, mobile apps, self-management, user experience

## Abstract

**Background:**

Diabetes management involves a large degree of data collection and self-care in order to accurately administer insulin. Several mobile apps are available that allow people to track and record various factors that influence their blood sugar levels. Existing diabetes apps offer features that enable integrations with various devices that streamline diabetes management, such as continuous glucose monitors, insulin pumps, or regular activity trackers. While this reduces the tracking burden on the users, the research highlighted several issues with diabetes apps, including issues with reliability and trustworthiness. As pumps and continuous glucose monitors are safety-critical systems—where issues can result in serious harm or fatalities—it is important to understand what issues and vulnerabilities could be introduced by relying on popular diabetes apps as an interface for interacting with such devices.

**Objective:**

As there is a lack of research examining in detail the integrations and potential suitability of apps as part of a wider self-management ecosystem, our goal was 2-fold. First, we aimed to understand the current landscape of device integrations within diabetes apps and how well they meet users’ needs. Second, we identified the key issues users of the most popular apps face currently and what features are the source of these issues.

**Methods:**

Through searches in Android and iPhone app stores, we systematically identified 21 diabetes apps that offer integrations. We conducted a detailed analysis of 602 user reviews. For each review, we recorded its sentiment, features and issues, and additional contextual information provided by the review writers. We used descriptive statistics to analyze the features and issues. We also analyzed the reviews thematically to identify additional trends related to the context of use and the consequences of issues reported by the users.

**Results:**

The reviews focused on key features that users found the most important, including device integrations (n=259, 43%), tracking (n=194, 32.2%), data logging (n=86, 14.3%), and notifications (n=70, 11.6%). We found that 327 (54.3%) of the reviews were negative versus 187 (31.1%) positive and 88 (14.6%) neutral or mixed, and the majority of reviews (n=378, 62.8%) mentioned issues. The biggest issues related to device integrations included inability to connect with external devices (n=95, 25.1%), inability to store, manage, or access data (n=49, 22%), unreliable notifications and alerts (n=35, 9.2%), issues caused by or related to software updates (n=31, 8.5%), hardware issues (n=24, 6.4%), and issues with accessing the app, related services, or associated hardware (n=12, 3.2%).

**Conclusions:**

Apps for diabetes management are a useful part of self-care only if they are reliable and trustworthy, reduce burden, and increase health benefits. Our results provide a useful overview of desired features for diabetes apps alongside key issues for existing integrations and highlight the future challenges for artificial pancreas system development.

## Introduction

### Background

Diabetes is defined as a group of disorders that share both hyperglycemic (hyper) and hypoglycemic (hypo) events caused by insulin insufficiency [1]. Hyperevents occur when blood sugar is too high, while hypoevents occur when blood sugar is too low. The 3 most common types of diabetes include type 1 diabetes (T1D), in which the pancreas does not produce insulin; type 2 diabetes (T2D), in which body cells become resistant to insulin over time; and finally, gestational diabetes, which occurs during pregnancy [2]. Due to the nature of the disease, diabetes involves a large degree of data collection and self-care in order to accurately administer insulin and avoid these hyper- and hypoevents. As a result, there is a large burden placed on individuals to track blood sugar levels, carbohydrate intake, activity levels, medications, insulin levels, and so on.

To assist with diabetes management, several technologies have been developed in order to help lower the burden for diabetics as well as to increase health benefits [3]. Since the 1990s, with the discovery of short- and long-acting insulin, insulin pen devices have been used to allow diabetics to better control their blood sugar levels [4]. Newer technologies have been developed, as access to technology has increased, such as continuous glucose monitors (CGMs), which allow for easier data collection on blood sugar levels, or insulin pumps for automatic scheduled delivery of insulin. Access to these relatively low-cost devices has also led to do-it-yourself (DIY) artificial pancreas (AP) systems, which use a combination of these medical devices with software systems such as web or mobile apps in order to create a closed loop between automatically taking blood sugar levels and administering insulin as needed [5].

Examples of AP systems include open-source projects like Nightscout [6] or OpenAPS [7]. Both systems have 3 key components: a CGM sensor to collect information on blood sugar levels, an insulin pump to administer insulin, and a place to log information, such as a website or mobile app [6,7]. The exact devices and ways in which this information is used are up to the end user who is modifying the out-of-warranty devices. Interviews and trials with patients, care providers, and families demonstrate reduced levels of anxiety or fear and better health outcomes [8-10]. In addition, patients and parents are asking for devices that reduce their burden so that diabetes has little impact as possible on their everyday life [11,12].

While DIY AP systems are an ad hoc but useful solution to a serious issue, the necessity for them has been created due to the slow and lengthy processes required for developing safety-critical medical technologies [13,14]. While authors highlight the benefits of the devices, there is also discussion around the need for adequate regulation of commercially available AP systems in addition to collaboration between industry, care providers, and patients to ensure that the developed systems are fit for purpose [15,16]. For example, Drew [17] highlighted that from the DIY movement, at least 1 open-source algorithm has been approved by the US Food and Drug Administration; however, it can be difficult to make devices from different manufacturers work together, causing interoperability issues.

To software engineers, these issues are not surprising, as in their essence, AP systems are internet of things (IoT) systems, combining multiple actuators and sensors in order to deliver a product. As such, the key challenges that occur in IoT systems—such as connectivity issues or device and data privacy [18]—may also apply in the context of integrating different diabetes devices and apps. For AP systems, in particular, we can see these issues occurring in the above DIY solutions. This is further compounded by the fact that the management of each type of diabetes is different, and while some elements are common, management for specific individuals is also unique, requiring a complex set of system requirements [19].

To help address this issue, several diabetes apps have been developed to serve as a way for end users to interpret their data and make informed decisions about insulin dosage. These apps can work with a combination of devices as part of an AP system or alternative care approach. Wu et al [20] reviewed how diabetes apps contributed to lifestyle changes and demonstrated that they are effective for T2D. Conversely, Zhang et al [21] found in their survey that the use of apps was higher among patients with T1D than T2D [22]. Key features for diabetes apps include but are not limited to medication management, blood glucose management, physical activity, diet and nutrition, and weight management [23]. Performance expectancy of these functions and social influence are key factors in patients regularly engaging with the apps [24]. In addition, concerns related to the ethics and reliability of apps have been raised by Huang et al [25], where only 1 app in their study was able to meet all 8 criteria for trustworthiness. This highlights the need for further investigation of the apps themselves as a key part of AP systems and their use in managing all types of diabetes. However, despite an extensive search of existing literature, to the best of our knowledge, there are no papers that explicitly explore users’ perceptions of diabetes apps in relation to device integrations.

### Objectives

It is evident that mobile apps are becoming an integral part of diabetes self-care and management. However, while several studies have demonstrated their potential health benefits [20-22], to the best of our knowledge, there are no studies that examine in detail the integrations and potential suitability of diabetes apps as a collective to determine if they meet user needs and function appropriately as part of a wider AP system. As stated previously, social influence has a large impact on whether or not apps are used, along with reliability and trustworthiness being key factors in uptake [24,25]. There are also challenges in functionality and use being determined by different types of diabetes as well as the unique requirements for each individual and the different situations they find themselves in [19]. Therefore, there is a need to better understand the current landscape of device integrations and how well they meet users’ needs.

As user reviews are a rich and useful source in exploring user feedback and needs for health-related apps (this is a common approach often used to examine a wide range of health apps, eg, focused on mental health [26,27], medication adherence [28,29], or diabetes [30]), the primary aim of this research was to investigate the reviews of diabetes management apps to determine wider themes and identify which functionality is important to users. We anticipate that as a result of the challenges that IoT systems like APs face, app reviews will highlight and reflect these challenges while also demonstrating which features are vital for end users’ self-care and diabetes management.

Furthermore, with AP systems and the widespread use of DIY solutions to self-care, exploring integrations and connectivity with other devices, such as insulin pumps, CGMs, and smartwatches, will be important to ensure that systems work as expected. Due to the safety-critical nature of the diabetes management apps (safety-critical here meaning that a flaw in the system could lead to severe injury or even fatalities), it is crucial to ensure that systems are safe to use. Therefore, the secondary aim of this research is to identify the key issues users of the most popular apps face at the moment and what features are the most common sources of these issues. The primary contributions of this paper are (1) an in-depth investigation of user perceptions of diabetes apps and their associated integrations and (2) a summary of key features and issues with diabetes app functionality that will impact AP systems in the future.

The rest of the paper is structured as follows: first, we begin with the methodology of our study outlining the data collection procedure, including inclusion and exclusion criteria as well as keywords used in our search. Next, we present the key findings of our search, highlighting that the majority of reviews expressed a negative sentiment and providing insights into features common in diabetes apps in addition to issues with integrations. This is followed by the Discussion section, where we describe the principal findings, implications for AP systems, and limitations of our study, before finishing with concluding remarks.

## Methods

### Overview

Following the procedures informed by similar app reviews [26], we decided to first identify relevant apps and then focus on their reviews. As users tend to post extreme reviews (either very positive or very negative [31]), public reviews are a great way of understanding factors that are important to users and identifying the most pressing issues. At the same time, by focusing only on reviews mentioning specific functionality, we can reduce the potential bias that the tendency to post extreme reviews could introduce. Our data collection, extraction, and analysis process are described in detail below.

### Data Collection

We used the New Zealand and the UK versions of Google Play to identify apps and reviews for analysis. We focused on Android apps predominantly, as Android phones are generally more affordable to the wider population and account for 70% mobile market share [32], which helps to capture the majority of users. However, while in both the United Kingdom and New Zealand, the market share of Android devices is closer to 50% [33,34], there is high duplication of apps across the Google Play and Apple App stores, with the most popular apps available on both platforms. Furthermore, Google Play reviews are easier to access and process for reviewing, as the Apple App Store does not allow copying of content.

**Figure 1.**
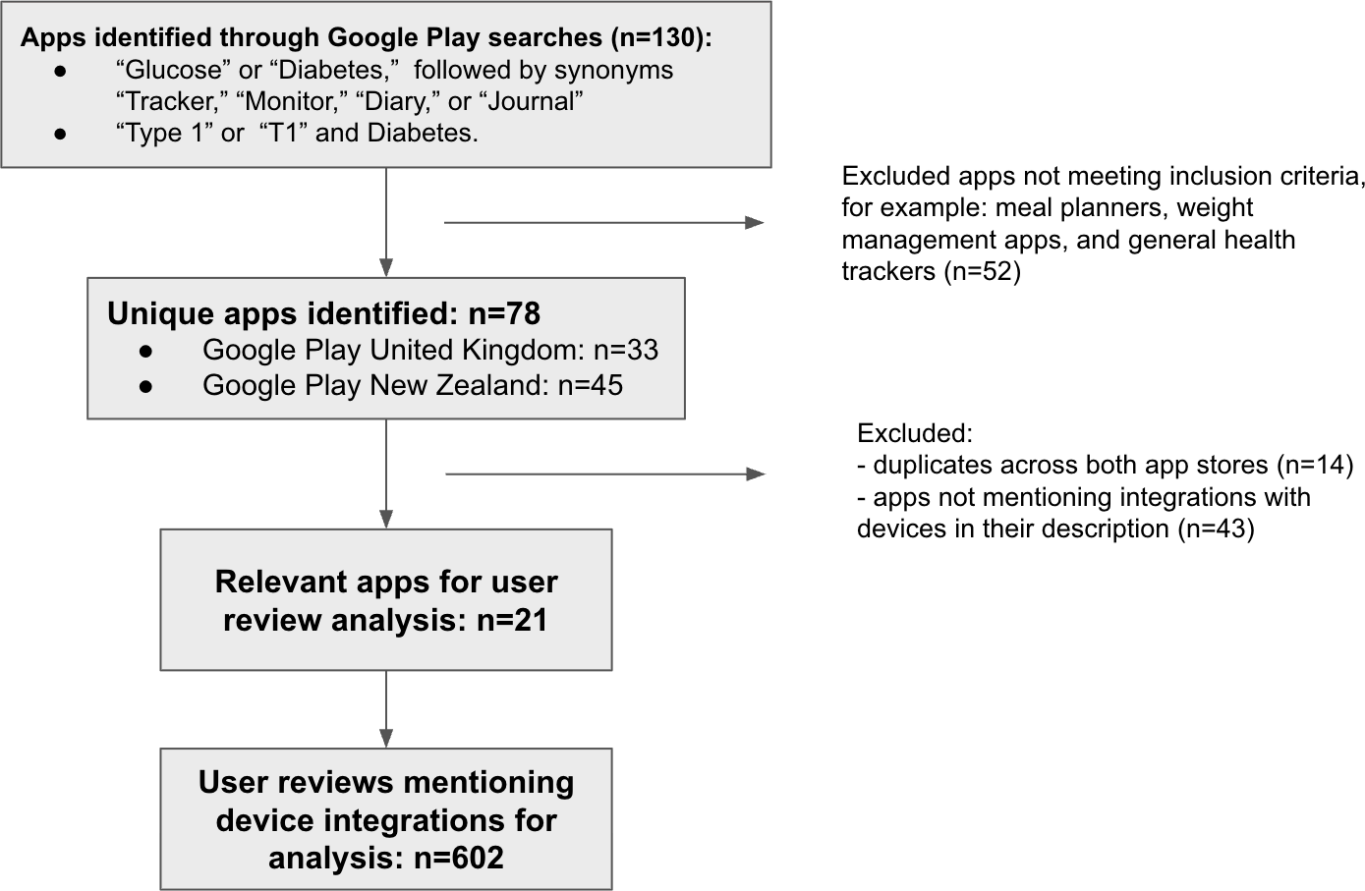
An overview of the data collection process.

Figure 1 summarizes our data collection process. First, to identify relevant diabetes apps, we used the following keywords in the web version of Google Play: (1) “Glucose” or “Diabetes,” followed by synonyms “Tracker,” “Monitor,” “Diary,” or “Journal” and (2) “Type 1” or “T1” and Diabetes.

Our focus was to investigate apps that target core diabetes management activities such as tracking blood glucose levels and administering insulin, as they are more relevant to AP closed-loop systems. As a result, we included T1D as part of our keywords instead of T2D or gestational diabetes. Furthermore, there are several apps that aimed at T2D like the Type 2 Diabetic Cookbook [35] or Pregnant with Diabetes [36], which focus their functionality according to these conditions but do not provide functionality for diabetes management in general.

The searches resulted in a total of 130 apps, with 28 apps available in both the New Zealand and the UK Google Play app stores. Each author checked the descriptions of the apps to identify those that specifically mentioned device integrations (ie, apps that could connect and import data from other devices, eg, CGM, pumps, and wearable activity trackers), which resulted in 21 relevant apps. These apps and their functionality are summarized in Multimedia Appendix 1. Note that all the apps used in this study had both Android and Apple versions. Each app selected was also available in a wide variety of countries.

Next, for the subset of these 21 apps mentioning integrations, we downloaded user reviews during October and November 2023. The reviews had to meet the following inclusion criteria: (1) mentioned specific functionality or features. Reviews that simply provided praise (“Great app!”) or criticism or complaints (“This app is awful!”) were excluded, (2) published in the last 12 months, and (3) posted in English.

Given that the number of reviews varied across the apps, reaching over 26,000 for some popular apps such as OneTouch Reveal, we decided to limit the total number of downloaded reviews per app to 50 to avoid skewing the analysis toward features or issues characteristic to only a few popular apps (an approach informed by prior research [28]). Limiting the number allowed us to have a broad range of reviews across all apps.

When an app was available in both app stores and had features or content characteristic to this location, we collected reviews for both versions, for example, FreeStyle LibreLink – NZ and FreeStyle LibreLink - GB were treated as separate apps due to the limited number of reviews in the New Zealand app store. We believe this occurred because access to technologies is impacted by government funding, for example, CGM funding is still under review in New Zealand [37], while it is readily available in the United Kingdom [38] (although access to certain devices is limited). This allowed us to include popular apps that may have been unnecessarily affected by external factors such as funding schemes.

The reviews were manually copied from a desktop version of Google Play by the authors. In total, we downloaded 602 relevant reviews for 21 apps; 1 app (SocialDiabetes) did not have any reviews that met the inclusion criteria.

### Ethical Considerations

This work did not require an ethics application for several reasons. First, app review information is publicly available and published on the relevant app store, as a result, these data may be viewed by anyone with internet access. Furthermore, there is no direct interaction with participants, and sensitive or private information collected. Usernames were also omitted to help ensure reviews, and consequently, individuals are not identifiable.

### Review Annotation and Analysis

Next, we divided the reviews equally between the 2 authors. Each review was annotated by one author, and then the annotations were moderated by the other. For each review, we collected the following information: sentiment (positive, negative, neutral, and mixed), list of mentioned features, whether any integrations were mentioned (yes or no), whether any issues were mentioned (yes or no), details of any issues, and additional open-ended comments that summarized additional points covered in the review.

We used descriptive statistics to analyze the data and identify the main trends. We also analyzed all reviews thematically using the open-ended comments as initial codes. These comments were added during review annotation and moderation and were later reviewed and expanded by the authors. Through this process, we identified 11 feature categories that help to contextualize the findings in wider user experience (tracking, integration, data logging, notifications, accounts, user interface, data sharing, monitoring, compatibility, calculations, and data privacy) and additional concerns related to tracking others (often children) and general fear that unreliable apps can cause deaths.

## Results

### Overview

For the 602 reviews collected for diabetes apps, we were able to identify 11 feature categories (Table 1). Of the reviews collected, we found that over half (n=327, 54.3%) expressed a negative sentiment, while only 187 (31.1%) were positive. The remaining reviews were either neutral (n=15, 2.5%) or included mixed comments (n=73, 12.1%). Similarly, 301 (50.1%) reviews mentioned some type of device integration, and 62.8% (n=378) mentioned bugs or faults within the system. Detailed information about the 21 diabetes apps and reviews, including their version number, sentiment, features, integrations, and related issues, can be found in Multimedia Appendix 1.

**Table 1.** Feature categories mentioned in user reviews (N=602)^a^.

Features	Definition	Values, n (%)
Integration	The ability to connect with external devices.	259 (43)
Tracking	The ability to store data relevant to diabetes management.	194 (32.2)
Data logging	The ability to add contextual information in the app.	86 (14.3)
Notifications	All features related to alarms, alerts, reminders, and tips.	70 (11.6)
User accounts	The ability to log in or out of associated accounts in order to access app functionality.	38 (6.3)
User interface	Any information associated with the user interface.	34 (5.6)
Data sharing	The ability to share static data with an external source.	27 (4.5)
Monitoring	The ability to share dynamic data with an external source.	15 (2.5)
Compatibility	Hardware or software issues between devices.	15 (2.5)
Calculations	The ability to use the app to calculate bolus and other dosage levels.	9 (1.5)
Data privacy	How much control and freedom the user has over where their data are stored and who it is shared with.	8 (1.3)

a Some reviews mentioned more than 1 feature type.

### Diabetes App Features

Table 1 gives an overview of the feature categories identified in the diabetes app reviews. The most common feature mentioned was device integrations (n=259, 43%), that is, the ability to connect, gather, and share data from external devices such as CGM, automated insulin pumps, smart pens, smartwatches, and so on. Users also highlighted how collecting data from these devices allowed them to make informed decisions about their insulin levels and diabetes management. One user said in their review:

*FINALLY! A glucose app that actually works with my paired metre! I was considering buying a new metre and now I don’t have the need. Additionally, this app creates charts and reports with useful DETAILS! My metre’s companion app only shows daily numbers. I’m so happy I found this app!!*

[Glooko - Track Diabetes Data]

After integrations, tracking was the next most commonly mentioned feature (n=194, 32.2%). It referred to functionality that allows users to record data either automatically or manually to manage their diabetes. In the reviews we collected, users mentioned medication, carbohydrates, blood glucose levels, weight, and activity tracking as relevant to their management. In addition, trends from these data were also useful to allow users to make data-informed decisions on their diabetes management. One review stated:

*This is a great app for keeping track of your readings. I personally like the long-term charts which show trends and the times of your day that you have to pay a bit more attention to your sugar levels*.

[Diabetes:M - Blood Sugar Diary]

While tracking and data logging features are related, data logging allows users the ability to provide contextual information around the data that they are collecting. In total, 86 (14.3%) reviews explicitly mentioned data logging. Some users liked taking photos of meals or entering manual readings from test machines, while others put in feature requests for the ability to make notes related to specific readings, for example, one review said:

*... Please, I’m begging, give us a simple “Notes” section? I can write what exactly I ate, or that I accidentally skipped a meal/dose/whatever*.

[mySugr - Diabetes Tracker Log]

Mentions of notifications appeared in 11.6% (n=70) reviews and covered a wide variety of information within the apps. Some notifications were simply reminders for entering meal information and so on, while others were alerts or alarms notifying users that their blood glucose levels were out of range. Users found these notifications helpful:

*... The most useful for me are the ongoing notification & widget (both have trend arrows), it might seem simple but it really does help with trying to stay in range, also, a big plus is 3rd party apps allow you to see both ongoing notification & widget on a WearOS watch ...*

[Gluroo Diabetes Logger]

However, others were frustrated by the inability to control these notifications, especially when it related to scheduling and editing the alerts. User reviews highlighted several examples of how such uncontrollable alerts could affect everyday life and that the one-size-fits-all approach is not adequate. For example, one user stated:

*... Also, alarms override phone settings. Sitting in a wedding, or in church, you’ll get a loud alarm. Only way to stop it is to turn off your phone ...*

[Dexcom G7]

Many reviews were also quite emotional, highlighting users’ frustration and the need for reliable devices that simply work:

*Buggy, intrusive and a battery drain. Pairing is slow, connectivity is poor and readings are inaccurate. Sensors fail before even finishing warmup. Alarms blare all day and all night reporting false lows and connectivity alerts, even with my phone on vibrate or “Do Not Disturb.” I was forced to turn all Dexcom notifications off in the system settings. This is a bad, dangerous app, clearly designed by someone who has never had to actually wear the sensor. People will DIE using this GARBAGE app*.

[Dexcom G7]

Issues with the lack of control were also extended to other parts of the app. For example, another review described issues faced by shift workers:

*When are you going to fix being able to adjust the hours of meals & sleep time out of the normal 9 to 5 hours for people that work a graveyard shift. App doesn’t let you set your sleep time to be during the day, for people that have to work a graveyard shift, so it totally makes the app useless. Here we are a year later!!! and you haven’t addressed my issues. not all people live a 9 to 5 life*.

[OneTouch Reveal]

When user accounts were mentioned in reviews (n=38, 6.3%), they were usually in reference to issues with access. That is, users either had trouble registering or logging into an existing account. For example:

*Had app before but it kept crashing at launch. Uninstalled it. Reinstalled recently. I keep getting stuck at login. It will not let me sign up or login. Uninstalled*.

[One Drop: Better Health Today]

Comments directly related to the user interface appeared in 5.6% (n=34) of the reviews. Data visualization in the form of graphs and charts was important to end users so that results were easy to interpret and follow. Furthermore, many reviews highlighted users’ resistance to interface changes, which were often seen as undesirable. For example, one review stated:

*Was good until the last release, when the daily Time in target was removed, and the 24 hour rolling graph. Both my key measures were removed! Please bring these back*.

[FreeStyle LibreLink - GB]

Similarly, the ability to share data with external sources was mentioned in 4.5% (n=27) reviews. For example, sharing an app-generated report with a doctor or nurse was considered useful:

*I love this app it makes it so easy for me to show my doctor my progress my high and my lows because you carry your phone everywhere it’s so accessible.*

[iHealth Gluco-Smart]

Some apps provided users with the ability to not only share data but also monitor other users. For example, the ability for a parent to monitor a child’s blood glucose levels while at school allows them to be kept informed of their time-in-range. Of the reviews collected, 2.5% (n=15) explicitly mentioned monitoring features:

*It’s a blessing. It was very difficult to record and track my mother’s diabetes. It made it so easy. Thank you so much for this app. I would really recommend it*.

[forDiabetes: diabetes tracker]

Surprisingly, one user mentioned in their review using one of the apps to monitor their cat, as the app synced well with their device and worked with custom ranges:

*Simple diabetes tracking app. Just had to setup a custom range for my cat. I set 80‐120 for before a meal and 80‐300 after a meal for a generic range which I received from a vet. Works with alphatrak3 for pets*.

[Blood Sugar Diary for Diabetes]

Compatibility was also mentioned in 2.5% (n=15) of reviews. While some issues were minor, such as phones no longer being able to access an app, others highlighted interoperability and cost issues in accessing diabetes technology:

*My device is only compatible with the Clarity app and is incompatible with both the G6 and G7 CGM apps. I love using the Dexcom but the receiver is extremely expensive and inconvenient. Please add the OnePlus Nord N300 5G to the list of Dexcom accessible devices soon. Thank you!*

[Dexcom Clarity]

Diabetes calculations were mentioned less frequently than initially anticipated (only n=9, 1.5% of reviews). We suspect this is because regulatory bodies such as the US Food and Drug Administration or Product Safety Australia have recalled apps, where these calculations have failed due to input errors [39,40]. However, users expressed that they hoped these calculators would come back as they found them useful, even though they knew why they were removed. For example, one user mentioned:

*Best logging and calculating app I’ve found. Not perfect, but quite good. It was the only bolus calculator available until that feature was withdrawn due to U.S. regulations. Sad to see that gone*.

[Diabetes:M - Blood Sugar Diary]

Similarly, data privacy was only explored in 1.3% (n=8) of the reviews collected. However, of those who did mention data privacy, permissions and how their data were shared, accessed, and used were important:

*Consent for Private Data Collection Required to Use the App - No option to decline = Uninstalled*

[SmartLog]

Another user highlighted that the app continued to run in the background despite not being given the appropriate permissions to allow this functionality:

*I do not give this app permission to run in the background, yet it continues to do so. I have to force quit it to get it to stop*.

[mySugr - Diabetes Tracker Log]

### Issues With Integrations

Given that the majority of reviews were either negative or mixed (ie, covering both good and bad features or experiences), it was no surprise that several issues were identified. In general, 378 (62.8%) reviews mentioned some issues, including 280 (46.5%) reviews focused specifically on issues related to device integrations. Table 2 summarizes the integration issues found in the user reviews.

**Table 2.** Integration issues mentioned in user reviews (n=378).

Issue	Definition	Values, n (%)
Connectivity	The inability to connect with external devices.	95 (25.1)
Data	The inability to store, manage, or access data.	49 (22)
Alerts	The inability to notify users of important messages.	35 (9.2)
Updates	The inability to update the software and associated hardware.	31 (8.5)
Hardware	Hardware that does not respond as expected.	24 (6.4)
Access	The inability to access the app, services, and associated hardware.	1 (3.2)

One of the most common issues found in the user reviews associated with integrations was the inability to connect with external devices. Several reviews discussed user’s difficulty with connecting with medical devices such as insulin pumps or CGM in order to manage diabetes levels. One user mentioned:

*When it works, it’s really great, but it seems to struggle to work properly, plagued with signal losses. I’m not sure if it’s the sensor or the software at this point. My friend’s app also loses signal from a sensor on her arm to a phone in her pocket* ...

[LibreLinkUp]

As highlighted in the previous review, connectivity issues also led to issues with the ability to store, manage, or access data. One of the primary benefits of the apps is to allow users to make data-informed decisions about their diabetes management. However, when data are not collected correctly, this leads to a lack of trust in apps:

*The app leaves much to desire. No charts showing your readings so you can visually get an idea of what to expect out of your blood sugar trends. The tester is cheap junk. I’ve cross checked it multiple times with the most accurate tester on the market and sometimes it’s over 70 mg/l higher. If I wasn’t double checking between both testers, I very well could have taken a lethal dose of insulin. Absolutely do not recommend for more unstable type one diabetics like myself. It’s honestly awful*.

[One Drop: Better Health Today]

Further compounding these issues were alerts failing to notify users when their blood glucose levels were no longer in their optimum range. Delays caused by unreliable software or connection issues could be potentially dangerous, especially when a prompt remedial action was required:

*Rarely has updated numbers, alerts can come after kid has been low for an hour*.

[LibreLinkUp]

Software updates could also cause significant problems for users, sometimes causing them to be unable to continue using their system as expected:

*Decided to stop working all of a sudden, will NOT reconnect with Bluetooth at all. Big stuff up on updates. Not good for people who need the software*.

[SmartLog]

The above issues could have more serious consequences when the app was used on behalf of someone else. While there were just a few reviews describing such use (n=15, 3.9%), they highlight potential risks, as it is often vulnerable users who are monitored this way. Some of these reviews mentioned connection issues, data loss, and general lack of reliability, often caused by app updates:

*This app has lost its credibility after the recent update (Sep 27, 2023). There’s frequent signal loss, and the BGL doesn’t update for as long as 1 hour! Alarms don’t trigger in time. Many times, I received a low alarm after successfully treating a potential low. I hope the developers understand how crucial this is! My child is only 3YO, which makes it so important to be able to monitor her levels, even when she’s not with me. It was a near flawless app before this update. Possible to roll back?*

[Libre LinkUp]

Alongside these software problems, issues with the hardware devices themselves appeared in the user reviews. From problems with hardware not responding to issues with getting readings, it was clear that hardware reliability was crucial to the success of an app:

*I’m on my 2nd 90-day G7 shipment. I’ve had one or two sensors fail before their expiration time & at least one that kept dropping its signal in each batch. I’m using the back of the arm, washing it with soap and water, & wiping my arm with alcohol and drying before inserting the sensor as instructed. I love the greater freedom that CGMs provide & Dexcom has been great about replacing bad sensors, but they need to improve reliability…*

[Dexcom G7]

Finally, access was a key issue mentioned in 3.2% (n=12) of reviews. This could be related to an inability to log into an account but also included features hidden behind paywalls, causing accessibility and equity concerns:

*I purchased this app assuming it’d allow me to track and then share information with my physician. It would - if I agreed to pay an additional $60.00 per year for access to reports. I've submitted a request for a refund to google; I’m not buying an app just to be told to pay for a subscription. That’s absurd*.

[Diabetes & Diet Tracker]

The reviews mentioning the above issues also highlighted the need for good customer service. Many reviewers mentioned unresponsive developers when complaining about the various factors, which could further reduce the trust in the apps:

*Would be a great app if the built in data imports worked. Until I can bring my data over I can’t use this. Contacted support and still no contact 3+ weeks later. Appears to be a pointless app. Rechecked, imports still not working*.

[Diabetes:M - Blood Sugar Diary]

## Discussion

### Principal Findings

#### Overview

The aim of this study was to investigate the role device integrations play in the use of smartphone apps for diabetes management. Our results show that apps are used for a wide variety of reasons to assist with diabetes management. When working as expected, they are highly valued and useful to ensure that users remain “in range” and improve health outcomes. However, when issues occur, users highlight how this could have serious and dangerous impacts on their overall health—particularly in relation to insulin administration, as too much or too little can trigger adverse events with negative health effects. In the following sections, we discuss the implications of these findings and contribute to a better understanding of issues related to using popular diabetes apps as part of AP systems.

#### Reliability, Trustworthiness, and Burden

Given the high number of negative and mixed reviews, it is evident that current apps do not adequately meet users’ needs. In particular, issues occurring either with hardware, such as CGM sensors, or the software itself would cause users to not trust apps to accurately track their glucose levels. Some reviews even highlighted that this would cause users to stop using the app altogether, while others talked about measuring themselves in multiple ways to ensure that they had accurate results. One of the fundamental goals of these apps is to assist with self-care management and to consequently reduce the burden; however, by forcing users to measure and track themselves in multiple ways, the apps reviewed have failed in this regard.

In addition, once users see that the app is failing, for whatever reason, they tend to uninstall the app and look for an alternative. That is, once trust is lost, it is difficult to regain. This is reflective of other results related to software trustworthiness with health apps. For example, Wicks and Chiauzzi [41] highlight that while there is great potential for health-based apps, a lack of regulation and adequate verification processes means that apps do not behave as expected, leading to poor quality and a lack of trust.

As the internet became a prevalent place for information sharing, the Health on the Net Code was created as a code of conduct in the health or medical domain for sharing information on websites [42]. Huang et al [25] adapted the code for diabetes medication management apps and found that only 1 app of the 143 investigated met all criteria. Our results are reflective of this, in that user reviews show some apps to be unreliable and therefore untrustworthy. This emphasizes the need for appropriate regulation and verification processes to ensure that apps are fit for purpose and meet users’ needs. Furthermore, as apps become part of AP systems and diabetics are more reliant on this technology, trustworthiness, reliability, and quality assurance will become increasingly important.

#### IoT Challenges Related to Diabetes Apps and Devices

As stated previously, IoT systems come with inherent challenges for many different reasons. Lohiya and Thakkar [18] identified 12 key challenges for IoT, 6 of which are reflected in the reviews analyzed, namely, connectivity, device monitoring and sensing, device and data privacy, data analytics, security, and evolution of IoT devices. Below, we explore these 6 key challenges in relation to the reviews analyzed.

Connectivity issues in IoT systems relate to multiple devices being connected to the network, while device monitoring and sensing relates to sensors being able to respond to real-time data. In the reviews analyzed, which mention integrations specifically, users were often working with only 1 sensor (such as a CGM) being connected with 1 app; however, despite this simple setup, users still encountered significant issues in tracking real-time data. In addition, there were multiple data losses, which meant that users could not trust the information they were receiving from the system.

Device and data privacy refers to the accessibility of data or devices being shared only as the owner of that data allows. While some user reviews explicitly mentioned privacy concerns, it was surprising to find that there were more issues around an inability to share data with others, such as doctors or caregivers, in a useful and readable format. This was linked with the data analytics challenge, in which raw data are processed into understandable information. Despite privacy issues and the prevalence of dangerous data access permissions in diabetes apps [43], users appeared to be less concerned with data privacy than we anticipated and more concerned with the meaning that they could infer from that data for their diabetes management to share with others. This can be a serious issue, as data collected by diabetes apps can be considered sensitive, which can introduce potential vulnerabilities if that data are stored on external servers—as is the case with most health apps [44]. This also exposes users at risk of data leaks and other security vulnerabilities [45-47].

Security is a challenge for IoT systems, as devices must be able to communicate and share information within specific networks, and there already exist interoperability issues across different vendors and devices [48]. This is also the case for diabetes devices, for example, with a lack of compatibility between sensors and CGMs from different manufacturers unable to communicate. Given that commercial gains are at stake, it is unlikely that a unified ecosystem will develop in the near future, although DIY diabetes projects such as OpenAPS can work with different types of CGMs [7]. The above issues and challenges are reflected in the diabetes apps reviewed, we found that there were significant issues with access and updates that relate to this challenge, which echo similar issues noted for other health apps, for example, users of mental health apps reported being “devastated” after an app update wiped out their historic data [26]. Access issues often prevented users from being able to log in and retrieve their information from apps. Similarly, updates as part of the usual software maintenance process could lead to new issues being introduced. Security will be an ongoing issue for diabetes technology, as it is inherently sensitive information shared over the internet. In fact, even people directly involved in diabetes DIY using Nightscout see it as high risk and requiring expert involvement [49]. Therefore, there is an opportunity for apps developed by experts to support this type of AP solutions. However, as highlighted by the reviews, it will be important to ensure that prioritizing security will not impede access to the data itself.

The last IoT challenge we encountered in the reviews was an evolution of IoT devices, or rather interoperability challenges, when a lack of standard design makes it difficult to ensure devices are compatible with each other. This was evidenced by the number of different hardware systems mentioned in the reviews, such as CGM sensors and other testing devices. As DIY systems become more prevalent, app developers will need to ensure that they are clear about device compatibility for the systems they develop.

#### Implications for AP Systems

As mentioned previously, AP systems use a combination of a CGM sensor, an automated insulin pump, and a website or smartphone app to create a closed-loop system that mimics a real-life pancreas [5]. As a result, the desirable features and challenges outlined in the user reviews—related to both the hardware and the app itself—will be relevant to AP system developers and the wider DIY community.

There are significant issues around the reliability, trustworthiness, and burden that smartphone apps create for diabetics. However, when they work well, they can lead to great health benefits as outlined in the reviews analyzed. AP systems that use apps like the ones investigated here will inherit the same issues if similar engineering processes are followed. Furthermore, the implications of inaccurate data on patient health and decision-making can negatively impact the user’s health. This is compounded by the incorporation of artificial intelligence algorithms, which are used to decide what level of insulin to administer. Without reliable data, the AP system may deliver an incorrect dosage, leading to hypo- or hyperglycemic events. Ghassemi et al [50] emphasize the importance of rigorous validation for these systems to ensure the work as expected and avoid bias. It is evident that good quality assurance practices will be imperative to the success of AP systems to ensure that they are reliable and trustworthy and meet users’ needs.

All current and proposed AP systems work by integrating a smartphone app as part of the closed-loop system. As phones are regularly updated, users must keep up with new app versions, changes to interfaces, and the cost barriers for newer phones. Similarly, older devices may struggle with battery life, which further impacts the use of older technologies as part of the AP system. This coupled with the regular maintenance required for pumps, and a continuous supply of CGM sensors significantly increases the burden on the end user. The need for user-friendly designs that are supported across multiple platforms and devices is necessary to ensure equitable access to the majority of diabetics.

Furthermore, as AP systems are themselves IoT systems, they inherit the challenges of IoT in addition to the issues outlined in the app reviews. As with most IoT systems, interoperability will be a key factor in their success. In the apps reviewed, hardware issues were mostly in relation to the single sensor and a single smartphone app—in contrast, AP systems may be far more complex, requiring communication between multiple sensors, pumps, and apps, leading to more potential problems. Therefore, the engineering of AP systems is an interesting and important avenue for future work.

### Limitations

The user reviews of diabetes apps were used in this research to determine whether apps meet user needs and how integrations are used. A limitation of this study is that we only investigated reviews published within the last year that explicitly mention features or functionality. There may be other themes that could have been discovered over a longer time period. Furthermore, we were unable to collect demographic data about reviewers, as they are anonymized. However, our data still give a good understanding of recent issues and features available in diabetes technologies, and the issues identified would occur regardless of the background of the user, which reduces the impact of this limitation.

Our focus on the app store means that our results reflect apps that are commonly available and attached to supported integrations. As a result, we do not investigate DIY solutions, as reviews are less readily available for these apps. Furthermore, there is the potential for bias toward extremely positive or negative comments [31] or toward more popular apps that receive significantly more user reviews. However, by limiting the number of reviews per app (by following an approach reported by Stawarz et al [28]) and focusing on reviews that explicitly mention features and functionality, we were able to reduce these biases.

Finally, our focus on user reviews allowed us to explore a wide range of apps and gather user experience data that would otherwise be difficult to access. While in-depth studies of individual apps would provide stronger data, this would not give us the same breadth of coverage or allow for the evaluation of such a diverse set of integrations. We believe that this provides the best possible analysis for integrations and the implications this may have for AP systems. Future research could explore the detailed implementation of various integrations within specific apps to better understand related issues and devise approaches to addressing them.

### Conclusions

Using user reviews as the basis for analysis, this research shows that the use of smartphone apps in self-care and diabetes management is a suitable solution, which can have positive health benefits for users. However, it is imperative that due to their safety-critical nature, such apps are reliable and trustworthy and meet users’ needs in order to prevent and avoid adverse events. In addition, the apps must reduce the burden of self-care for diabetics to be appropriate for everyday use. The challenges and opportunities we have identified in the diabetes apps open up new avenues for research and provide warnings for those engineering AP systems to ensure that they do not inherit the same issues. Future work could include analysis of the apps themselves using a tool such as the Mobile App Rating Scale [51] to compare and contrast with our analysis here. In addition, we should explore the way in which AP systems are engineered and investigate interoperability issues, finding a balance between regulation and quality assurance practices.

## Supplementary material

10.2196/62926 Multimedia Appendix 1 Complete app and user review data with analysis.

## Abbreviations

AP: artificial pancreas
CGM: continuous glucose monitor
DIY: do-it-yourself
IoT: internet of things
T1D: type 1 diabetes
T2D: type 2 diabetes
